# Underwater Localization and Mapping Based on Multi-Beam Forward Looking Sonar

**DOI:** 10.3389/fnbot.2021.801956

**Published:** 2022-01-07

**Authors:** Chensheng Cheng, Can Wang, Dianyu Yang, Weidong Liu, Feihu Zhang

**Affiliations:** School of Marine Science and Technology, Northwestern Polytechnical University, Xi'an, China

**Keywords:** SLAM, multi-beam forward looking sonar, point cloud, grid map, underwater vehicle

## Abstract

SLAM (Simultaneous Localization And Mapping) plays a vital role in navigation tasks of AUV (Autonomous Underwater Vehicle). However, due to a vast amount of image sonar data and some acoustic equipment's inherent high latency, it is a considerable challenge to implement real-time underwater SLAM on a small AUV. This paper presents a filter based methodology for SLAM algorithms in underwater environments. First, a multi-beam forward looking sonar (MFLS) is utilized to extract environmental features. The acquired sonar image is then converted to sparse point cloud format through threshold segmentation and distance-constrained filtering to solve the calculation explosion issue caused by a large amount of original data. Second, based on the proposed method, the DVL, IMU, and sonar data are fused, the Rao-Blackwellized particle filter (RBPF)-based SLAM method is used to estimate AUV pose and generate an occupancy grid map. To verify the proposed algorithm, the underwater vehicle is equipped as an experimental platform to conduct field tasks in both the experimental pool and wild lake, respectively. Experiments illustrate that the proposed approach achieves better performance in both state estimation and suppressing divergence.

## 1. Introduction

AUVs (Autonomous Underwater Vehicles) have been widely applied to perform various complex underwater tasks such as resource exploration (Ohta et al., 2016), environmental monitoring (Williams et al., 2012; Barrera et al., 2018), underwater rescue (Venkatesan, 2016), and military operations (Hagen et al., 2005), etc. To satisfies the safety and reliability, AUVs should acquire accurate localization in underwater unknown environments.

To achieve this goal, Doppler Velocity Logging (DVL) and Inertial Measurement Unit (IMU) are fused with acoustic long baseline (Matos et al., 1999), short baseline (Vickery, 1998), and ultra-short baseline (Hao et al., 2020) to calculate the position of AUVs. However, these traditional methods have shortcomings regarding to error divergence. DVL measures the speed by integrating the acceleration and meanwhile further calculates localization from dead-reckoning, the final result may, therefore, contain cumulative errors; The method based on the acoustic baseline needs to arrange the equipment in the environment in advance; Therefore, it is essential to use a more robust and reliable method to solve above problems. On the other hand, SLAM enables AUVs to fuse sensor data and build a map of an unknown environment, while localizing simultaneously. So far, sensors applied in underwater slam include cameras, side-scan sonar (SSS), single-beam mechanical scanning sonar (SMSS), and multi-beam forward-looking sonar (MFLS).

Camera-based underwater SLAM estimates the ego-motion by extracting and matching features from adjacent images and optimizing the pose at the back-end (Kim and Eustice, 2015; Hong et al., 2016). Jongdae Jung et al. proposed a vision-based SLAM, where artificial underwater landmarks help visualize camera poses (Jung et al., 2017). Suresh et al. proposed a novel method for underwater localization using natural landmarks (Suresh et al., 2019). Sparse features were obtained via an onboard upward-facing stereo camera through water for underwater localization. Although the cost of the camera is low, vision-based underwater SLAM has significant limitations. The camera's detection range is close and can only work in a clean environment with good light.

Compare to cameras, sonar emits sound waves in single or multiple directions and obtains information about the surrounding environment by analyzing each echo's strength and return time. Sonar-based method is, therefore, the development trend of underwater SLAM (Wang et al., 2017; Wang and Cheng, 2020). Chen et al. proposed an RBPF SLAM algorithm to tackle the issues of scan distortion and data sparseness caused by the slow-sampling mechanical scanning sonar, by carefully designed a sliding window-based scan module (Chen et al., 2020). The formed scans are then fed into the modified RBPF to build a consistent grid-based map. Siantidis et al. described a SLAM system with a dead reckoning system and a side-scan sonar (Siantidis, 2016), which can compensate for the position drifts. Aulinas et al. proposed a feature-based sub-mapping SLAM approach, which considered side-scan salient objects as landmarks (Aulinas et al., 2010). However, the long scanning period is quite challenging to meet underwater real-time performance, as the return of the side-scan sonar and mechanical scanning sonar image is delayed.

Meanwhile, MFLS is becoming more and more popular in underwater perception because of its solid real-time performance, small size, and easy installation (Hurtós et al., 2014; Wright and Baldauf, 2016). Wang et al. proposed a novel approach for underwater SLAM using an MFLS for 3D terrain mapping tasks (Wang et al., 2019). Instead of repeatedly projecting extracted features into Euclidean space, they applied optical flow within bearing-range images for tracking extracted features and assumed these features are sampled from a Gaussian Process terrain map. Neves et al. introduced a novel multi-object detection system, which outputs object position and rotation from MFLS images (Neves et al., 2020). Pyo et al. proposed a novel localization method in shallow water, where localization is based on passive-type acoustic landmarks. Through modeling, the distance from landmark to MFLS could be calculated (Pyo et al., 2017). However, a complete occupancy grid map using underwater vehicles with MFLS is still missing.

This paper presents a methodology for the SLAM algorithm based on MFLS, by building an accurate occupancy grid map and providing an accurate estimation of AUV poses. The occupancy grid graph can be used for subsequent global positioning and path planning. The main contributions of the proposed algorithm are in two aspects. (1) Aiming at the slow processing speed caused by a large amount of MFLS image data, a method is proposed to convert the collected sonar image into sparse point cloud format data through threshold segmentation and distance-constrained filtering. (2) Based on the proposed method, the DVL, IMU, and MFLS data are fused, and then the RBPF-based SLAM method is used to generate an accurate occupancy grid map, and at the same time, the drift of the inertial navigation can be suppressed.

The structure of the proposed approach is as follows. Section 2 introduces the characteristics of the MFLS used in this article. The proposed SLAM method for underwater vehicles is detailed in section 3. The experimental results are shown in section 4. Section 5 presents a brief conclusion and section 6 is our future work.

## 2. Problem Description of MFLS SLAM

**Figure 1:**
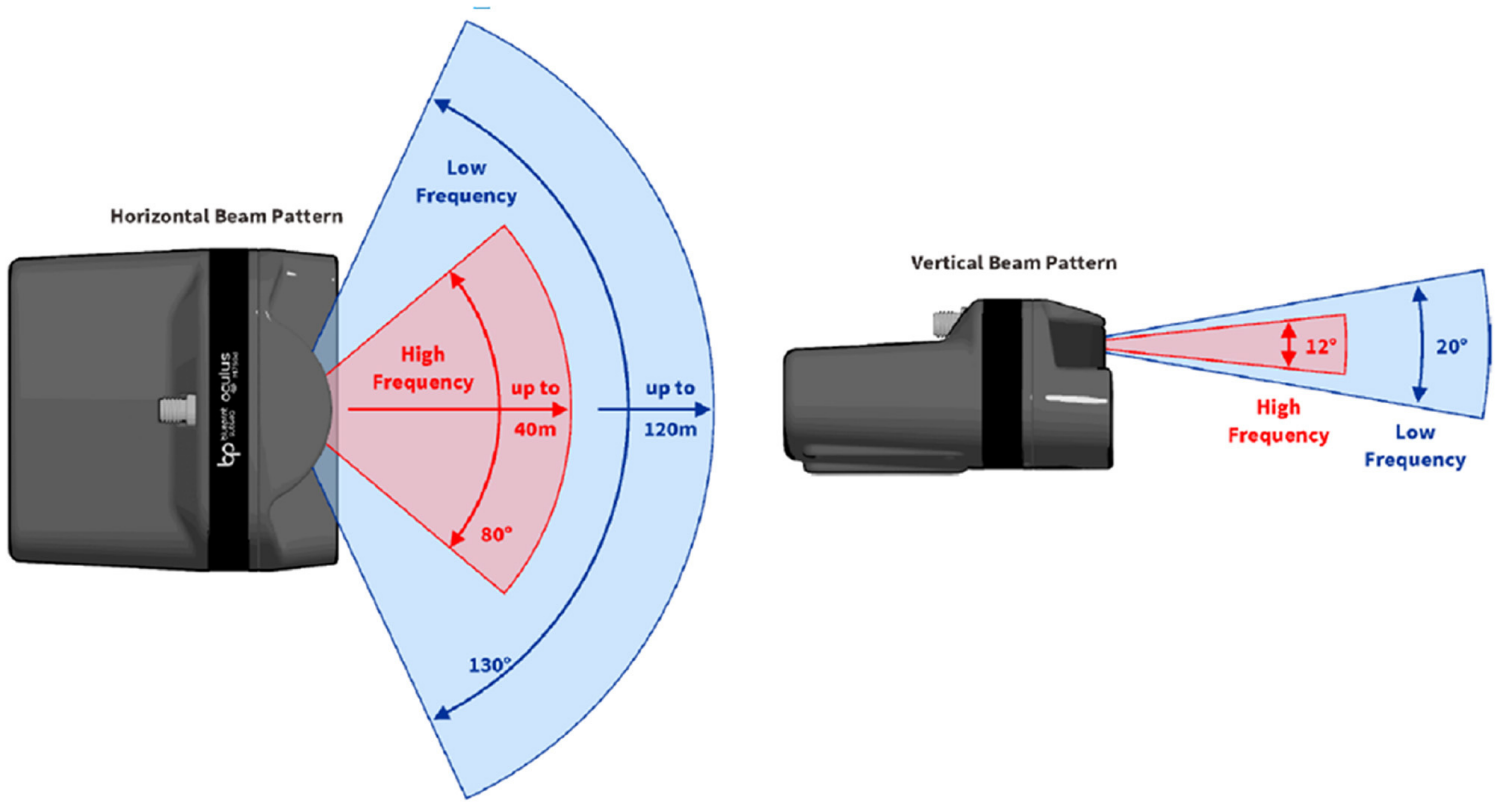
Schematic diagram of MFLS working mode.

**Figure 2:**
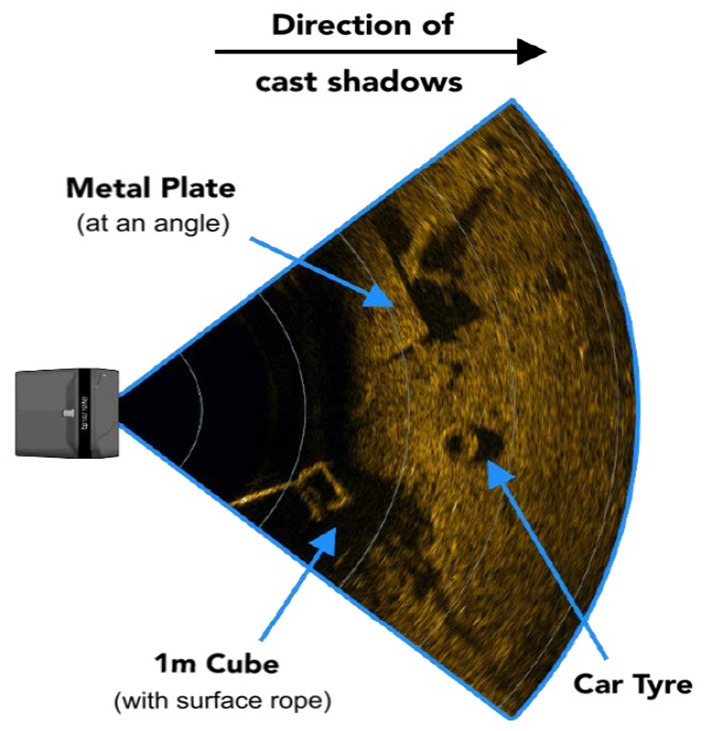
Example of scanning effect of 3 targets. The bright part indicates an obstacle with high echo intensity, and the dark part indicates that the echo intensity at that location is weak.

MFLS is an image sonar. It can emit multiple sound waves with a vertical width in the horizontal direction and detect the environment based on the echoes. The working principle is shown in Figure 1. However, it has no resolution in the vertical direction, so the result is a two-dimensional image. By measuring the flight time and intensity of the echo, images with different degrees of brightness will be obtained, as shown in Figure 2. The bright part indicates an obstacle with high echo intensity, and the dark part indicates that the echo intensity at that location is weak.

Generally, there are two MFLS data processing methods: image-level processing and echo intensity processing. However, due to the dense beams, high resolution, and the relatively large amount of image sonar data, processing directly from the image level will cause a large amount of calculation in the SLAM process, and it is difficult for the processor installed on the small AUV to process the data in real-time. In order to solve this problem, this paper converts the sonar image data into corresponding point cloud data and then uses distance-constrained filtering to extract the necessary information and reduce the amount of calculation. In the case of limited processor performance, the goal of real-time SLAM is achieved on a small AUV.

## 3. Proposed SLAM Method

**Figure 3:**
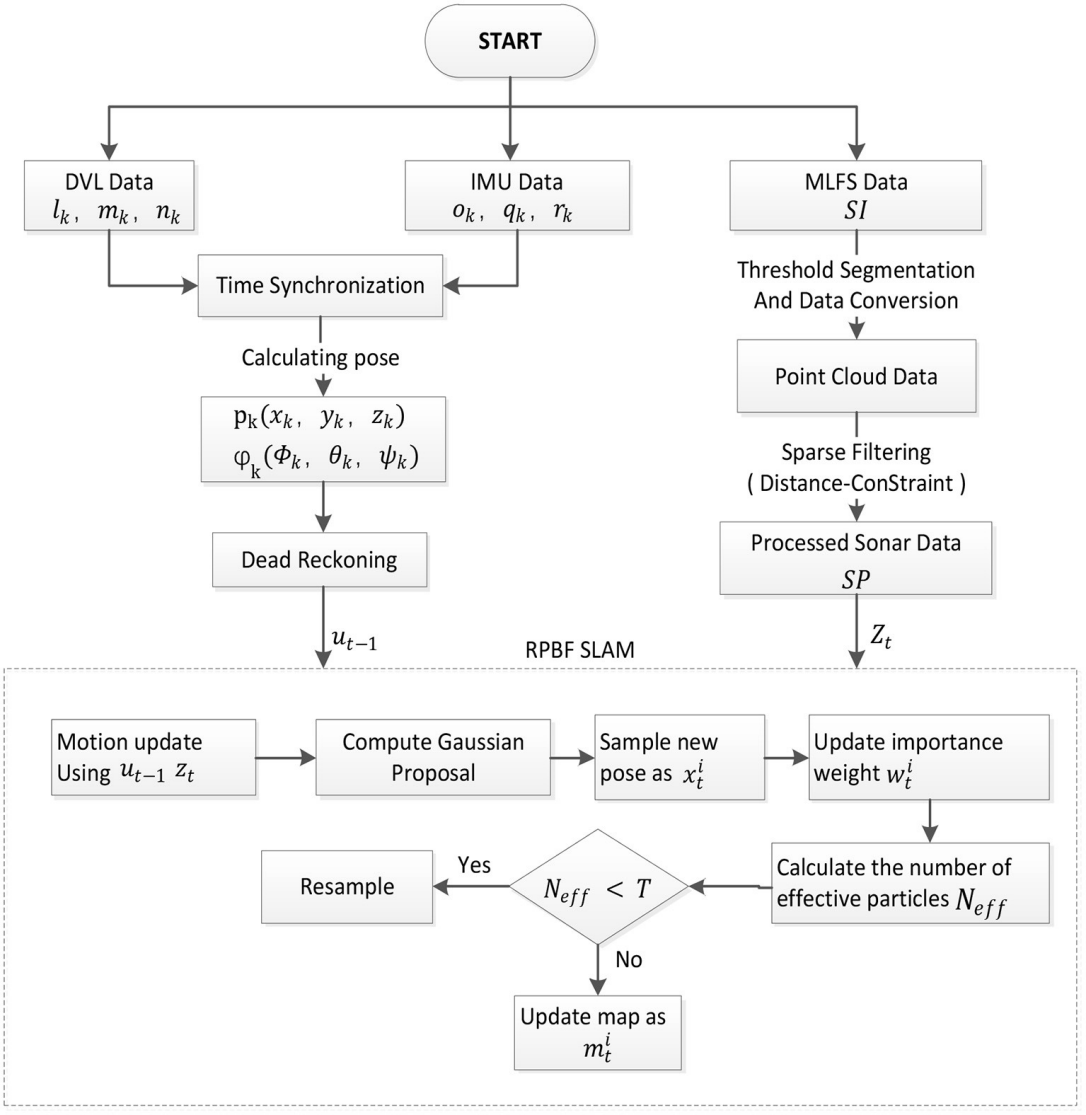
Architecture of the proposed SLAM algorithm.

Figure 3 is the overall framework of the SLAM algorithm proposed in this article. We first fuse DVL data and IMU data to obtain odometer data in the algorithm and use it for dead reckoning. At the same time, the MFLS sonar data is preprocessed. First, the sonar data is converted into point cloud data through threshold segmentation and data conversion. The obtained point cloud data is then subjected to sparse processing using distance constraints filtering. Finally, send the processed data into the RBPF-SLAM algorithm for positioning and composition.

### 3.1. Dead Reckoning

The function of the dead reckoning module is to apply the IMU and DVL to provide a rough estimate of the AUV pose. When the sonar samples the data, the dead reckoning module applies the extended Kalman filter (EKF) to estimate the attitude by fusing the data from these sensors.

Suppose the AUV estimated state is $X_{k}={\left[p_{k}^{T},\varphi_{k}^{T}\right]}^{T}$ in the global coordinate system at time *k*, where *p*_*k*_ represents the position of the AUV, and φ_*k*_ represents the attitude of the AUV. *p*_*k*_ and φ_*k*_ are, respectively, defined as


$$p_{k}={\left[x_{k}\;y_{k}\;z_{k}\right]}^{T},\;\varphi_{k}={\left[\phi_{k}\;\theta_{k}\;\psi_{k}\right]}^{T}$$

where *x*_*k*_, *y*_*k*_, *z*_*k*_ are the position coordinates in each axis in the global coordinate frame, and ϕ_*k*_, θ_*k*_, ψ_*k*_ are the Euler angles roll, pitch, and yaw in each corresponding axis.

Assuming that the linear velocity and angular velocity of the AUV are υ_*k*_ and ω_*k*_, they are jointly used as the control input $u_{k}={\left[\upsilon_{k}^{T},\omega_{k}^{T}\right]}^{T}$. Specifically, υ_*k*_ and ω_*k*_ are expressed as


$$\upsilon_{k}={\left[l_{k}\;m_{k}\;n_{k}\right]}^{T},\;\omega_{k}={\left[o_{k}\;q_{k}\;r_{k}\right]}^{T}$$

where each element in the two vectors is the linear velocity and angular velocity on each axis in the AUV coordinate system. Then the kinematics model of AUV can be expressed as


(1) $$\begin{array}{r} X_{k+1}=f\left(X_{k},u_{k}\right)=X_{k}+\Delta_{T}*J\left(X_{k}\right)*u_{k} \end{array}$$

where *J*(*X*_*k*_) is the transformation matrix, and Δ_*T*_ is the sampling time interval. *u*_*k*_ can be represented by DVL measurement value and IMU measurement value with Gaussian noise ω_*k*_ *N*(0, *Q*_*u*_). Due to this kind of noise, there will be error accumulation in dead reckoning. Therefore, other sensor information is called to correct the error during the update phase of EKF. Through formula (1), the AUV state can be estimated as


(2) $$\begin{array}{r} {\hat{X}}_{k+1|k}=f\left({\hat{X}}_{k|k},{û}_{k}\right) \end{array}$$

The covariance matrix used for the prediction error can be expressed as


(3) $$\begin{array}{r} P_{k+1|k}=F_{k+1}*P_{k|k}*F_{k+1}^{T}+G_{k+1}*Q_{u}*G_{k+1}^{T} \end{array}$$

where *F*_*k*+1_ and *G*_*k*+1_ are the Jacobian matrices obtained by solving the partial derivative of the nonlinear model function *f* about the state *X*_*k*_ and the noise ω_*k*_.

Finally, the model prediction is updated by applying the standard EKF update equation to generate the estimated pose of the AUV.

### 3.2. Threshold Segmentation and Data Conversion

The working principle of sonar is to generate an echo according to the sound wave encountering an object and then generate an image according to the time and intensity of the return of the echo. Due to water quality and acoustic interference, multi-beam sonar data will carry a lot of clutter and outliers in a natural environment. Direct conversion into lidar data for mapping will distort the resulting map. Therefore, it is necessary to filter according to the environment so that the data can better reflect the characteristics of the environment.

In this experiment, the raw sonar data were processed in three steps: threshold segmentation, data conversion, and distance-constrained filtering. The flow chart of the proposed algorithm is presented in Algorithm 1.

**Algorithm 1:**
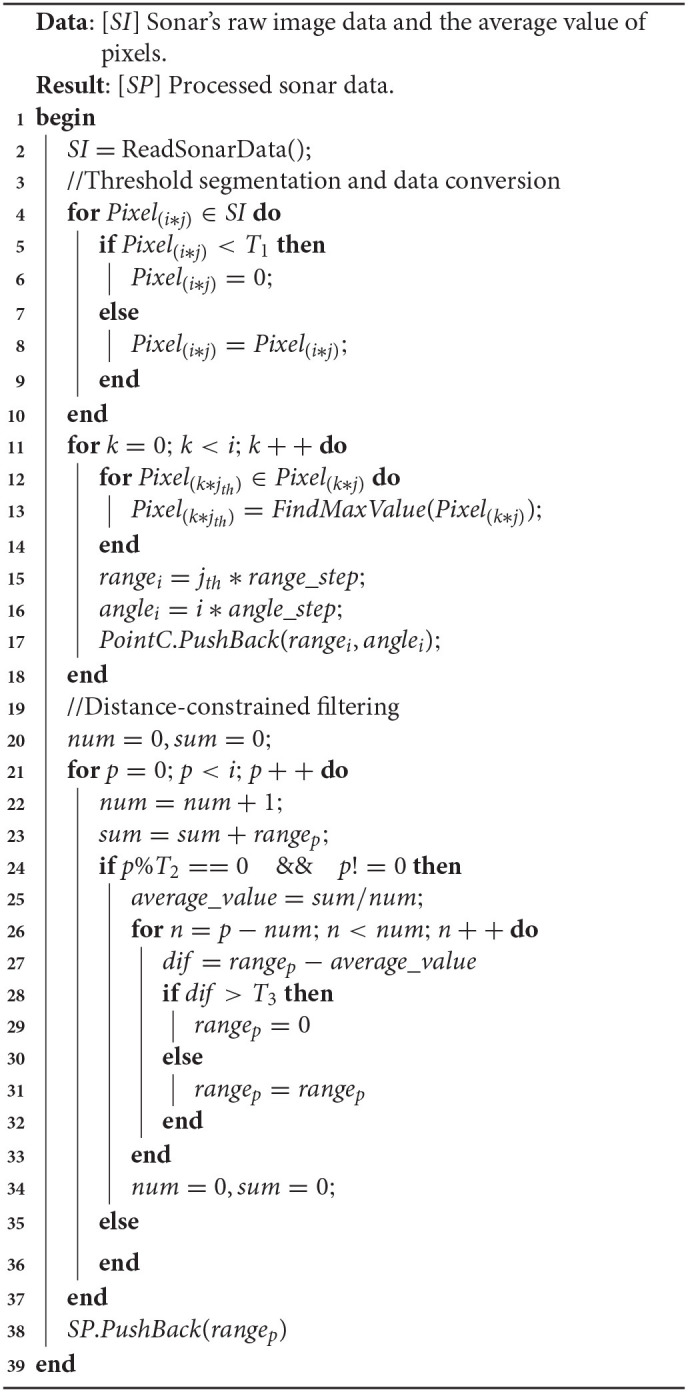
Sonar data process.

**Figure 4:**
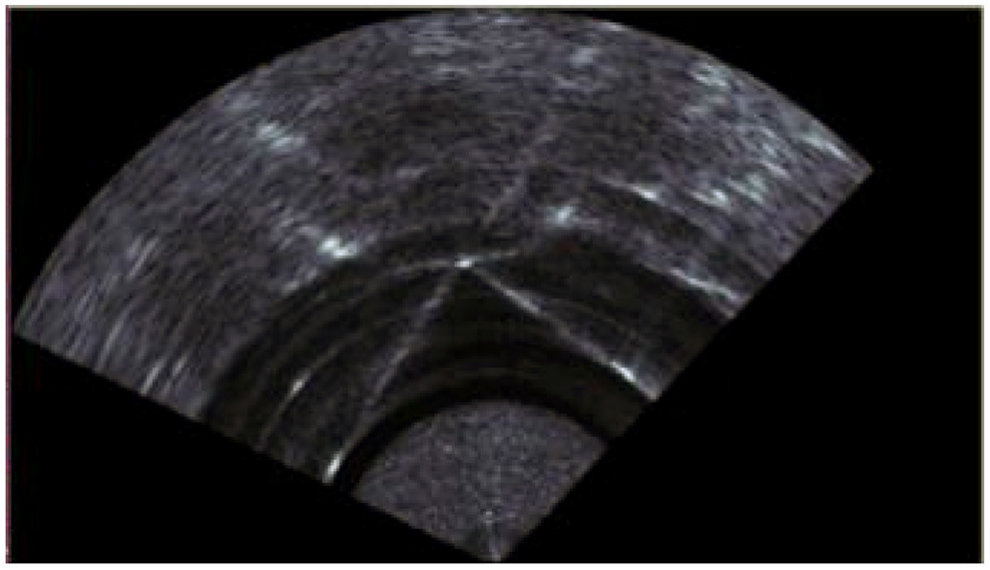
Sonar raw data. This is the raw data picture of the sonar facing the corner of the experimental pool.

*SI* is sonar's original data, an image generated with parameters with a scanning angle of 130° and a scanning distance of 40*m*. Figure 4 is an image generated by aiming at the corner of the pool with sonar, which contains a lot of clutter. *T*1 is the threshold for filtering selection. In general, we use the average pixel value as the threshold for filtering. At the same time, the threshold can also be manually set according to the water quality environment. Under normal circumstances, we put several targets in the water or look for an environment with apparent characteristics in the background. Then, we use sonar to scan in real-time and continuously adjust the threshold manually until the generated point cloud data can better reflect the target profile. At the same time, when watching open waters, less noise is generated, and the current threshold is selected as the optimal threshold in the current environment.

**Figure 5:**
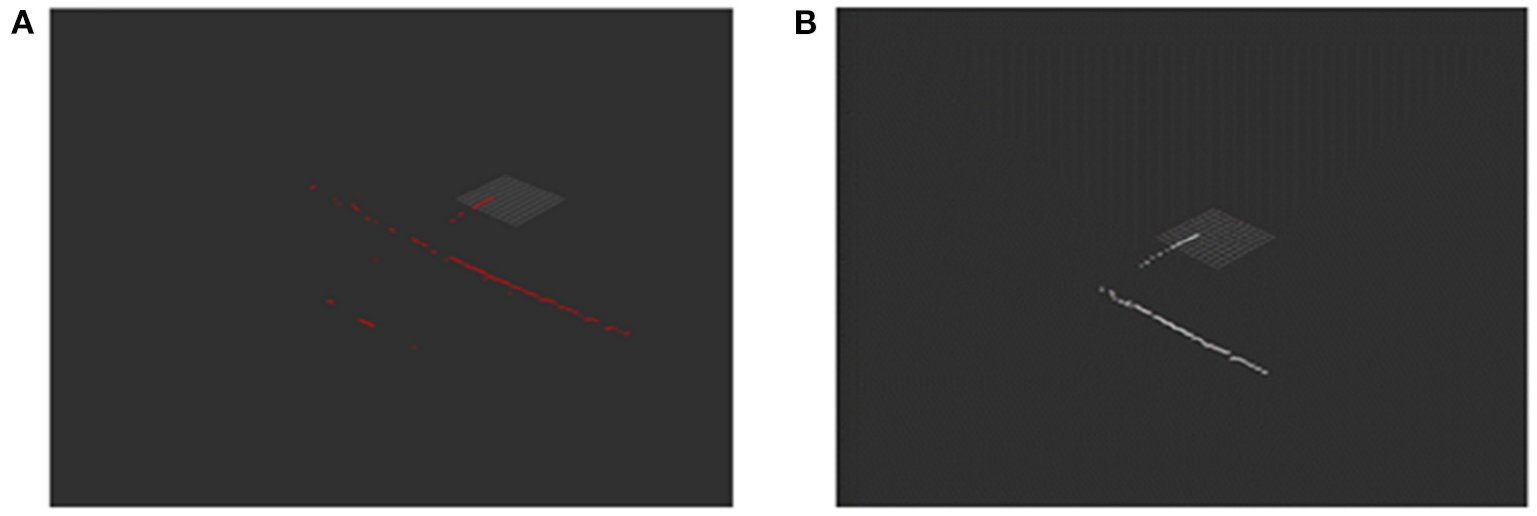
Panel **(A)** shows the point cloud data of the sonar raw data after threshold segmentation and data conversion. Panel **(B)** is the result of filtering based on distance-constrained.

The pixel value below *T*1 is assigned a value of 0, and the pixel value above *T*1 remains unchanged. Since only the features of the surface of the object are considered when constructing the map, we believe that the brightest point on the beam is formed by the sound wave hitting the surface of the object and returning. Therefore, we only select a point with the highest intensity on each beam as the target of interest. After preliminary filtering, we calculate the position of the target pixel in the sonar coordinate system according to the angular resolution *angle*_*step* and distance resolution *range*_*step* of the sonar. The resolution value can be changed by setting the sonar parameters. Calculating all the pixels of the sonar image, we can get the point cloud data, as shown in Figure 5A.

### 3.3. Distance-Constrained Filtering

From Figure 5A, we can find that many bright spots are generated behind the wall of the pool. This is not the data we want, and it will affect the positioning accuracy and mapping effect of the AUV. Aiming at these clutter interference, this paper uses a distance constraint-based method to filter out clutter while reducing the amount of data. Doing so can improve the real-time performance of the algorithm while ensuring positioning accuracy and mapping quality. The flow chart of this algorithm is shown in the lower part of Algorithm 1.

Process the obtained point cloud data *PointC*:

- Set a beam threshold *T*2, and divide all range data *range*_*i*_ into *A* = {[*range*_(0)_ − *range*_(*T*2−1)_], [*range*_(*T*2)_ − *range*_(2*T2−1)_].….[*range*_(*i* − *T*2−1)_ − *range*_(*i*)_]} according to the angle order(−65° −65°).
- Set the distance threshold *T*3, calculate the average of all *range*_*i*_ in *A*_*k*_, and then calculate the difference *dif* between each *range*_*i*_ and the average. If *dif* is greater than *T*3, the data is judged to be noise deleted, if it is less than *T*3, it is judged to be valid data.
- Save the valid data into the *SP*, and the sonar data processing ends.

Figure 5B is the point cloud image obtained after distance constraint filtering. It can be found that the clutter behind the wall of the pool is successfully filtered, and data that can truly reflect the environmental characteristics are obtained.

### 3.4. RBPF SLAM With MFLS

The flow chart of the proposed algorithm is presented in Algorithm 2. *PS* is the particle set generated according to the initial state of the AUV. According to the theory that the joint probability can be converted into the product of conditional probabilities, the solution of RBPF SLAM is to decompose the original SLAM problem into separate positioning and mapping parts (Grisetti et al., 2007).

**Algorithm 2:**
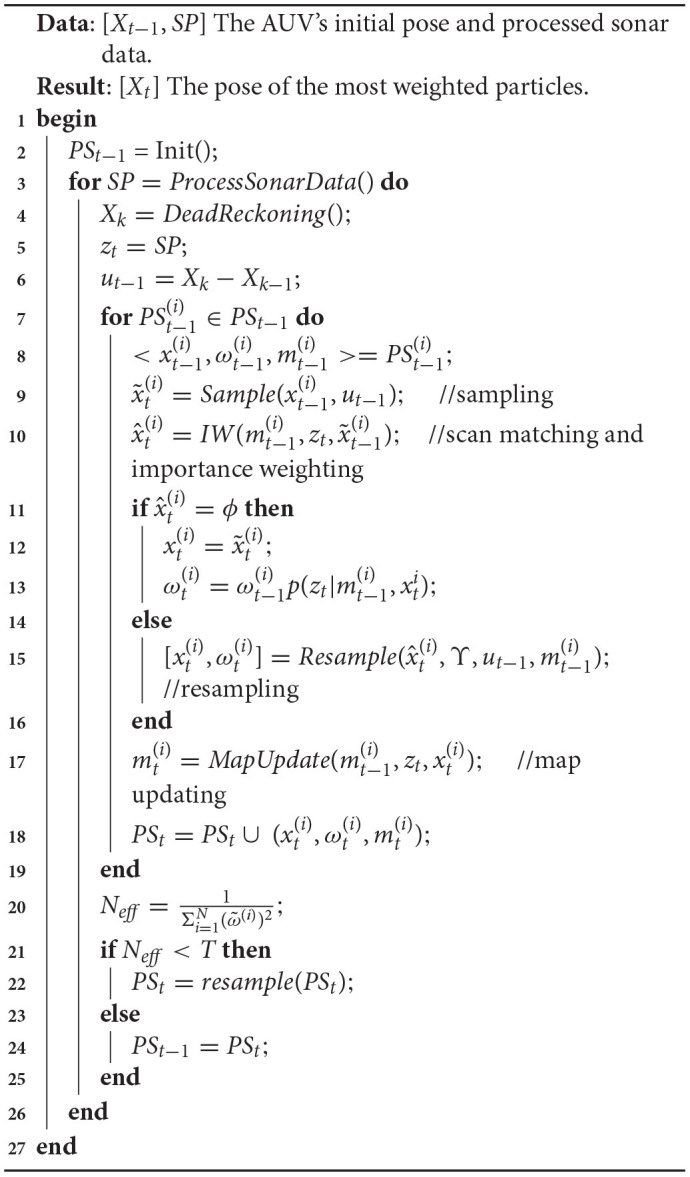
RBPF with multi-beam forward looking sonar.


(4) $$\begin{array}{r} p\left(x_{1:t},m|z_{1:t},u_{1:t-1}\right)=p\left(m|x_{1:t},z_{1:t}\right)\cdot p\left(x_{1:t}|z_{1:t},u_{1:t-1}\right) \end{array}$$

where *p*(*x*_1:*t*_|*z*_1:*t*_, *u*_1:*t*−1_) is the posterior of potential trajectories *x*_1:*t*_ given observations *z*_1:*t*_ and odometry measurements *u*_1:*t*_ of the AUV, *p*(*m*|*x*_1:*t*_, *z*_1:*t*_) is the posterior of maps, and *p*(*x*_1:*t*_, *m*|*z*_1:*t*_, *u*_1:*t*−1_) is the posterior of maps and trajectories. Given the values of *x*_1:*t*_ and *z*_1:*t*_, *p*(*m*|*x*_1:*t*_, *z*_1:*t*_) can be calculated analytically, so the key to the problem is to calculate *p*(*x*_1:*t*_|*z*_1:*t*_, *u*_1:*t*−1_).

To estimate the posterior *p*(*x*_1:*t*_, *m*|*z*_1:*t*_, *u*_1:*t*−1_), a group of particles is first introduced. Each particle is composed of the pose *x* of the AUV, the grid map *m*, and the weight ω. The particle filter algorithm incrementally uses dead reckoning values and sonar scan data to update the particle set. This process can be divided into four steps, *sampling*, *scanmatchingandimportanceweighting*, *resampling*, and *mapupdate*. Function ${\hat{x}}_{t}^{\left(i\right)}=IW\left(m_{t-1}^{\left(i\right)},z_{t},{\tilde{x}}_{t-1}^{\left(i\right)}\right)$ is a scan matching and importance weighting module, and its function is to calculate the pose that best matches the current map $m_{t-1}^{\left(i\right)}$ based on the current observation *z*_*t*_ and all samples ${\tilde{x}}_{t-1}^{\left(i\right)}$.

Then each particle is assigned a separate importance weighting based on the importance sampling principle $w_{t}^{\left(i\right)}$.


(5) $$\begin{array}{r} w_{t}^{\left(i\right)}=w_{t-1}^{\left(i\right)}\cdot \frac{\eta p\left(z_{t}|m_{t-1}^{\left(i\right)},x_{t}^{\left(i\right)}\right)p\left(x_{t}^{\left(i\right)}|x_{t-1}^{\left(i\right)},u_{t-1}\right)}{p\left(x_{t}|m_{t-1}^{\left(i\right)},x_{t-1}^{\left(i\right)},z_{t},u_{t-1}\right)} \end{array}$$

(6) $$\begin{array}{r} \propto w_{t-1}^{\left(i\right)}\frac{p\left(z_{t}|m_{t-1}^{\left(i\right)},x_{t}^{\left(i\right)}\right)p\left(x_{t}^{\left(i\right)}|x_{t-1}^{\left(i\right)},u_{t-1}\right)}{\frac{p\left(z_{t}|m_{t-1}^{\left(i\right)},x_{t}\right)p\left(x_{t}|x_{t-1}^{\left(i\right)},u_{t-1}\right)}{p\left(z_{t}|m_{t-1}^{\left(i\right)},x_{t-1}^{\left(i\right)},u_{t-1}\right)}} \end{array}$$

(7) $$\begin{array}{r} =w_{t-1}^{\left(i\right)}\cdot p\left(z_{t}|m_{t-1}^{\left(i\right)},x_{t-1}^{\left(i\right)},u_{t-1}\right) \end{array}$$

(8) $$\begin{array}{r} =w_{t-1}^{\left(i\right)}\cdot \int p\left(z_{t}|x^{\prime }\right)p\left(x^{\prime }|x_{t-1}^{\left(i\right)},u_{t-1}\right)dx^{\prime } \end{array}$$

Here, η = 1/*p*(*z*_*t*_|*z*_1:*t*−1_, *u*_1:*t*−1_) is the normalization factor produced by Bayes' rule where all particles are equal.

In function $Resample\left({\hat{x}}_{t}^{\left(i\right)},\Upsilon ,u_{t-1},m_{t-1}^{\left(i\right)}\right)$, the Gaussian approximation of the proposed distribution is calculated, and new particles are sampled for the next iteration based on the calculated result. Υ is the interval threshold for resampling in the vicinity area of ${\hat{x}}_{t}^{\left(i\right)}$.

Finally, the map $m_{t}^{\left(i\right)}$ is updated based on the estimated pose $x_{t}^{\left(i\right)}$ and the observed value *z*_*t*_. Then, select the map and pose of the particle with the largest weight among all the particles as the final constructed map and estimated AUV pose.

## 4. Experimental Result

Both simulation and practical experiments are conducted to verify the effectiveness of the proposed SLAM algorithm. Table 1 shows the main parameters of our algorithm for experiments.

**Table 1:** The algorithm parameters.

**Parameter**	**Value**
Map-update-intervel	0.3
maxRange	25
maxUrange	24
Number of particles	150
Resample threshold	0.5
Number of iterations	7

### 4.1. Simulation Experiments

In the simulation experiment, we used UUV-Simulator to create a Rexrov2 model, which is a full-propeller-driven ROV, and it is equipped with four cameras, four lights, and a wide range of sensors, including sonar, DVL, IMU, etc.

**Figure 6:**
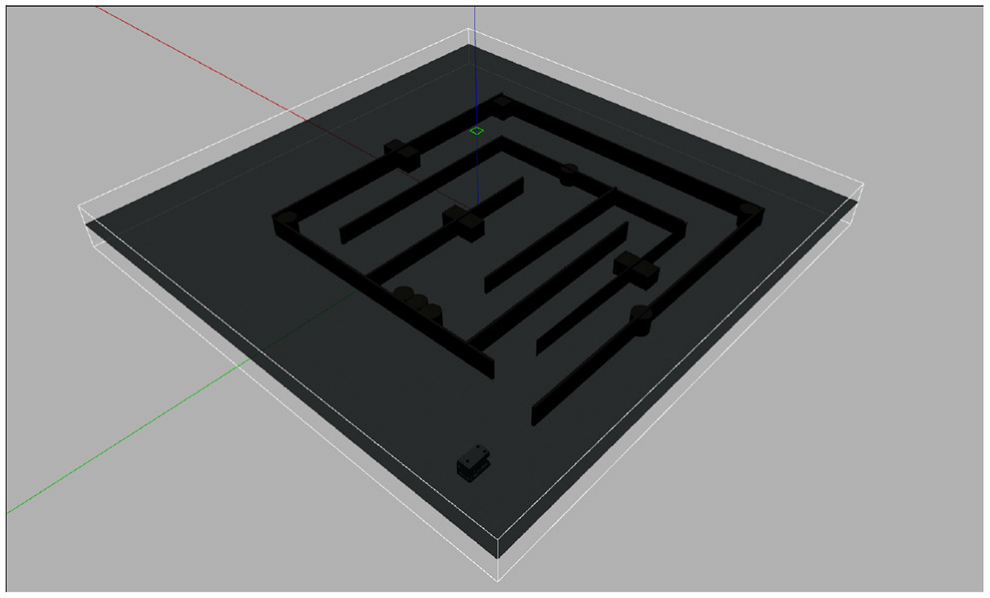
Experimental environment and Rexrov2 model.

Figure 6 shows the Rexrov2 model and simulation environment, an underwater maze. First, build a 3-D model of the environment and load it into the 3-D simulator Gazebo. To facilitate interaction with Gazebo, we used Robot Operating System (ROS) in our simulation. The drivers of IMU, DVL, and MFLS are loaded as Gazebo plug-ins and used to publish ROS-compatible data, which are subscribed by the proposed SLAM algorithm. Our ROV is controlled to navigate the maze for one round during the simulation. The algorithm's outputs include an occupancy grid map and an estimated ROV trajectory.

**Figure 7:**
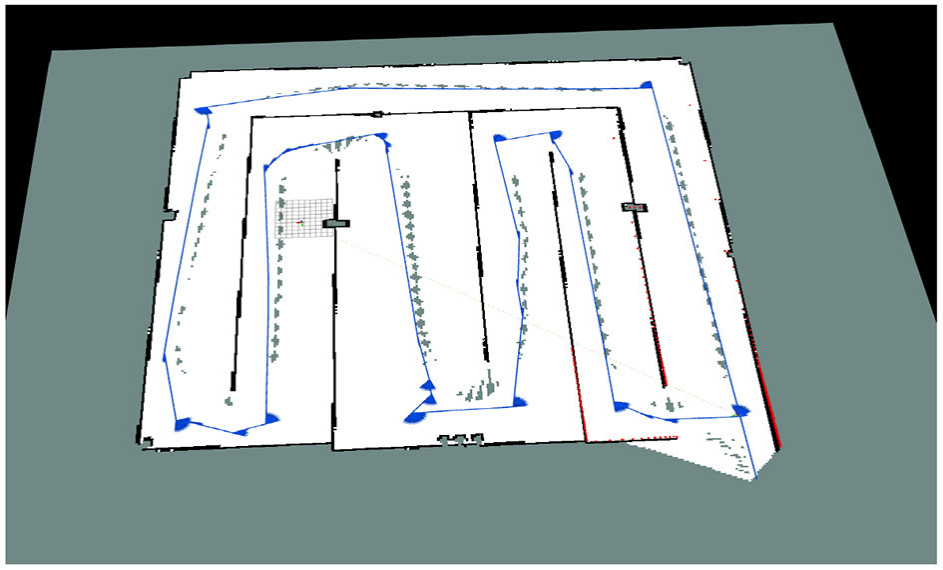
Two-dimensional grid map.

Figure 7 shows the occupancy grid map generated by the proposed SLAM algorithm. Based on this map, AUV can make path planning to avoid obstacles and reach the designated position.

**Figure 8:**
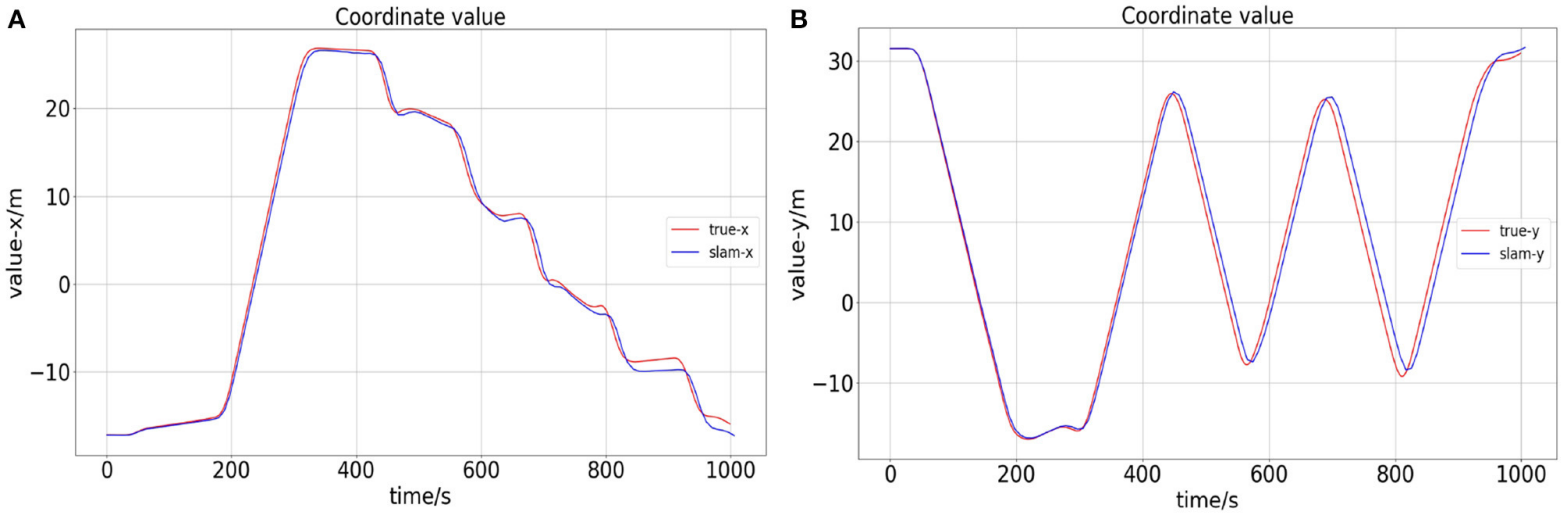
Panel **(A)** is the x-axis error between the calculated value of slam and the true value. Panel **(B)** is the y-axis error.

The comparison between the ROV position calculated by the proposed algorithm and the ground truth is shown in Figure 8. Their trajectory error is very small, and the simulation experiment proves the effectiveness of our proposed method.

### 4.2. Experimental Pool Experiment

**Figure 9:**
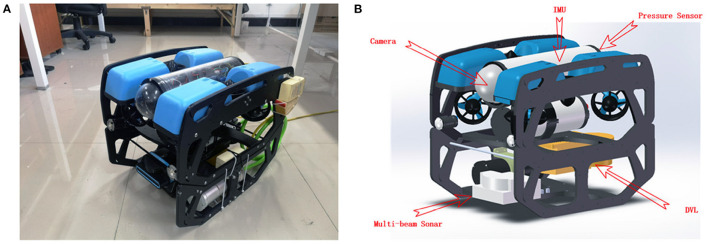
Panel **(A)** is modified BlueROV2, and Panel **(B)** is the sensor layout.

In the practical experimental, the open-source underwater robot platform BlueROV2 was used to complete the SLAM experiment of the underwater vehicle. In this paper, we only focus on the positioning and surveying indicators of the underwater vehicle, without considering the control. Therefore, the effect is the same regardless of whether the AUV or ROV is used as the experimental carrier to carry out the verification experiment. To make BlueROV2 meet our experimental requirements, we installed a multi-beam forward looking sonar and a DVL based on the original BlueROV2. Also, we installed a pair of power cat modules to achieve underwater BlueROV2 and the shore PC Long-distance data transmission. The modified BlueROV2 (as shown in Figure 9) can well meet the SLAM experimental requirements.

We first tested in the multi-purpose pool indoor. The size of the pool is 70 x 44 m. In the experiment, we controlled the BlueROV2 to face the wall for scanning and mapping.

**Figure 10:**
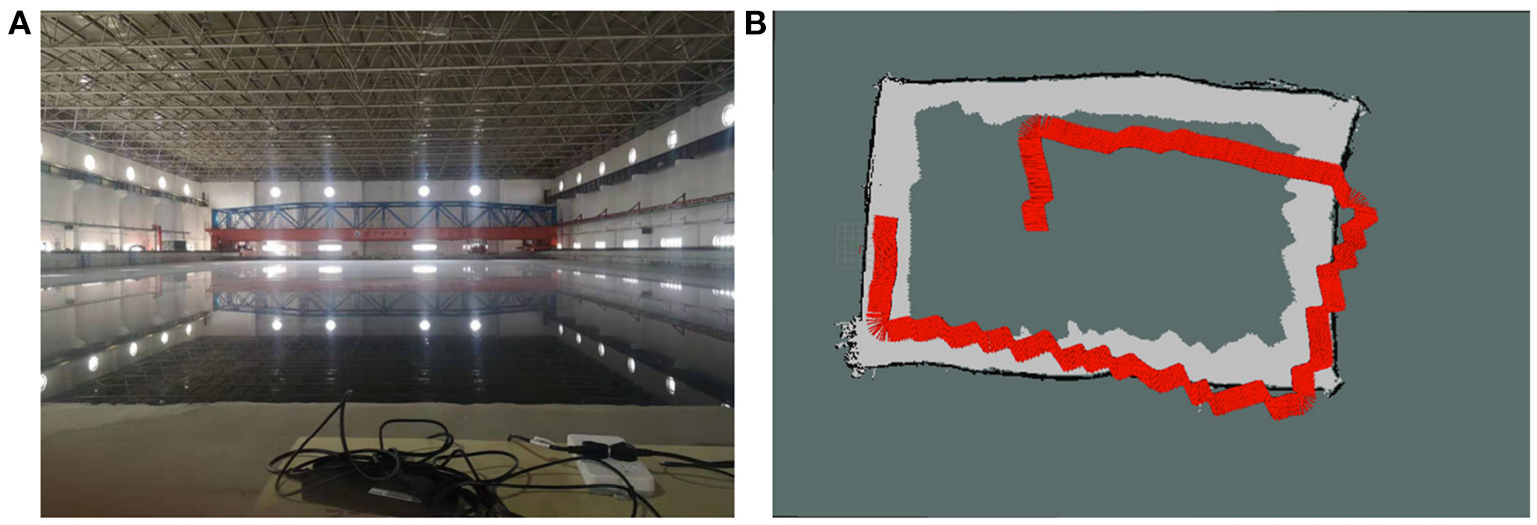
Panel **(A)** is the experimental pool environment. Panel **(B)** is the positioning and mapping effect of SLAM.

The experimental pool environment is shown in Figure 10A, and the positioning and mapping effect of the proposed algorithm is shown in Figure 10B.

In Figure 10B, the red part represents the trajectory of the odometer, the white part represents the detectable travelable area, the black part represents the obstacle, and the gray part represents the undetected area.

In this experiment, we used threshold segmentation and distance-constrained filtering to process the multi-beam sonar data and then used it to build the map. It can be seen from the mapping results that good results have been achieved. At the same time, it can be seen that the odometer error is continuously accumulating. When the scan is completed, the odometer has not completed closing, but the map established by the SLAM algorithm based on the multi-beam sonar has been closed, the odometer's position deviation is amended. Furthermore, the superiority of the SLAM algorithm based on multi-beam sonar used in this paper is proved.

### 4.3. Wild Lake Experiment

The field lake experiment was carried out in Liquan Lake, Xi'an, Shaanxi. During the experiment, we take a dinghy to approach the target environment, launch BlueROV2, and control it to scan the target environment for positioning and mapping experiments. Also, there is a GPS positioning antenna on the dinghy. When the BlueROV2 scanning environment, the boat closely followed BlueROV2 to obtain GPS coordinates near it, providing actual data for the quantitative analysis of the positioning surveying experiment.

**Figure 11:**
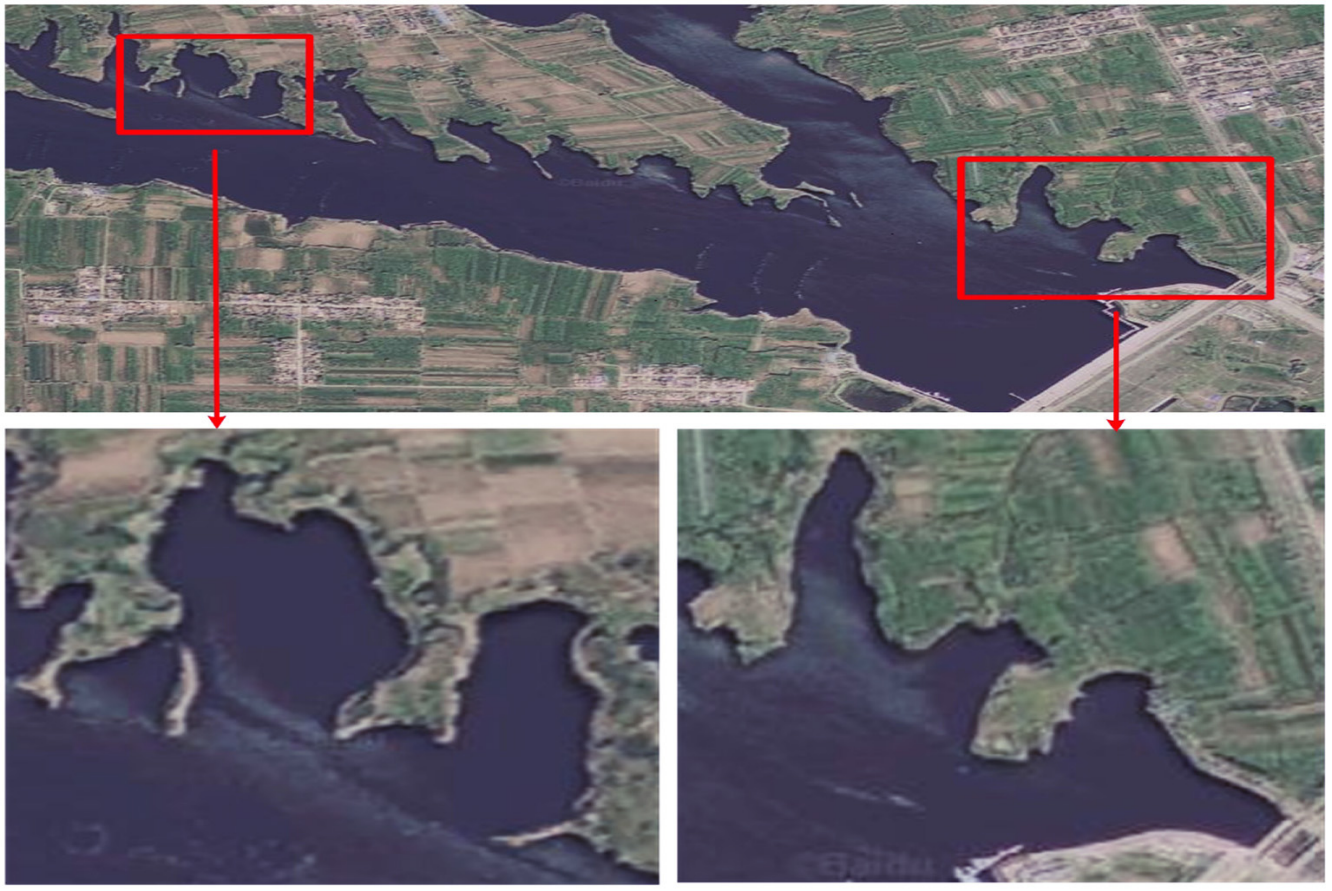
Satellite picture of the place where the experiment was conducted.

Figure 11 is a satellite image of the two experimental locations we choose.

**Figure 12:**
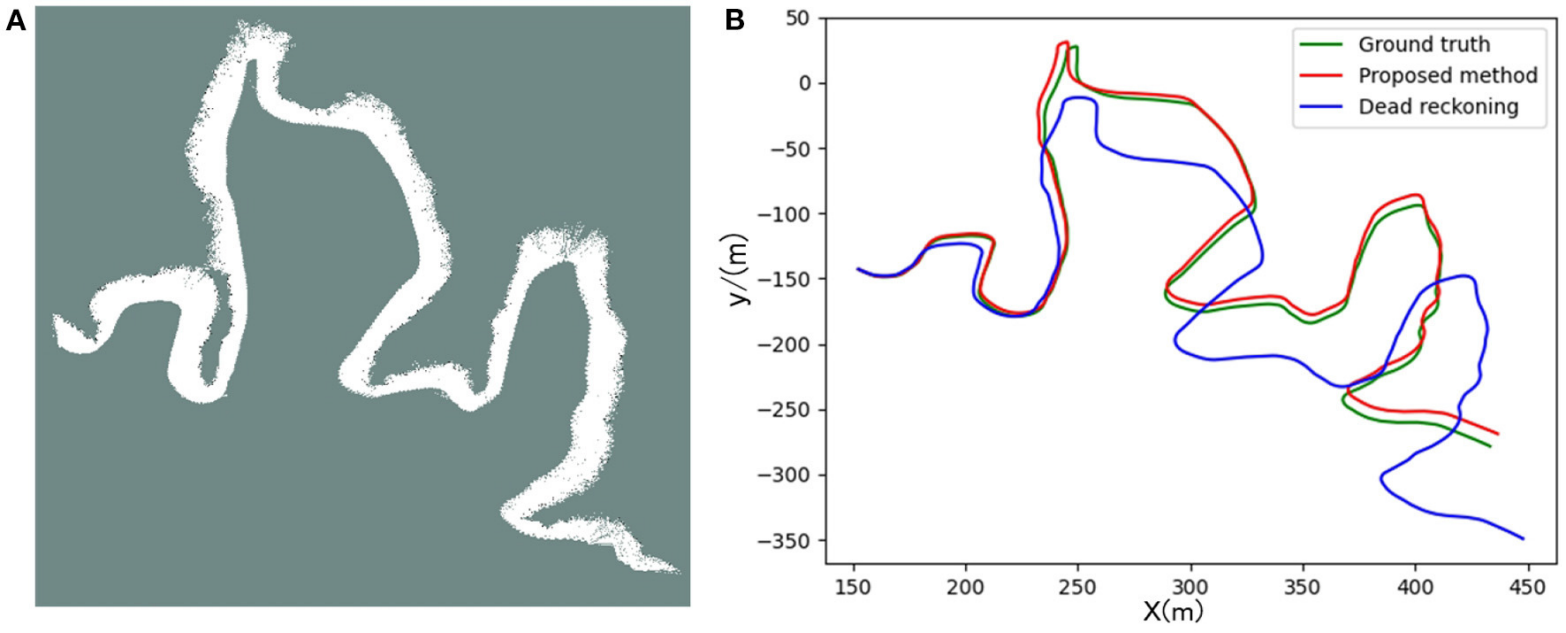
Panel **(A)** is the result of the two-dimensional grid map created in scene 1. Panel **(B)** is the trajectory calculated by the proposed algorithm and dead-reckoning, respectively.

**Figure 13:**
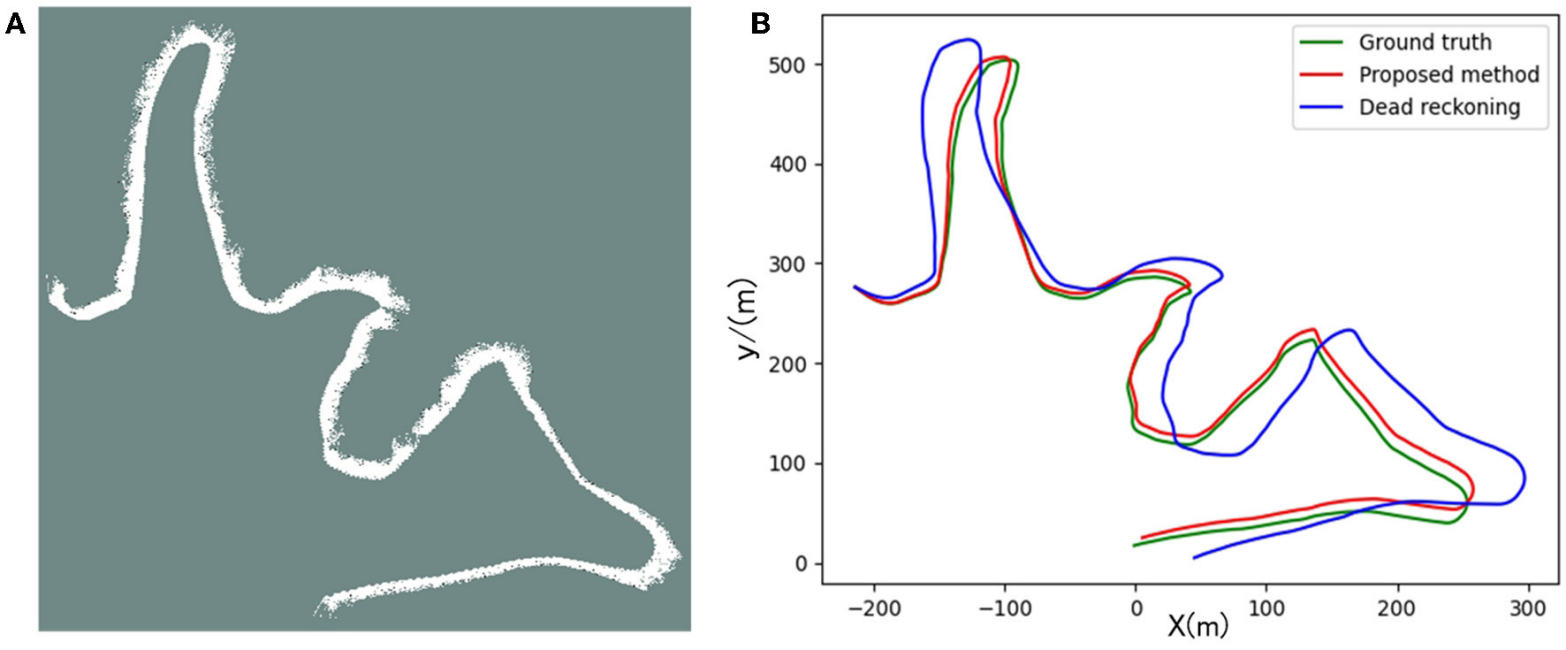
Panel **(A)** is the result of the two-dimensional grid map created in scene 2. Panel **(B)** is the trajectory calculated by the proposed algorithm and dead-reckoning, respectively.

Figures 12, 13 are the positioning and mapping results of the two experimental scenes with our proposed algorithm.

It can be seen from the experimental results (Figures 12A, 13A) that the mapping effect is mostly consistent with satellite images. There are several reasons for the deviation between the satellite image and the map created by SLAM:

- Due to changes in water level, satellite maps may deviate from actual maps.
- When BlueRov2 dives into the water, it scans the underwater extension of the lake bank, including objects such as branches and rocks. Unfortunately, the satellite image of this part of the underwater terrain is invisible, so the constructed map will be different from the satellite image.
- BlueRov2 cannot reach some waters, so the factual environmental information has not been thoroughly scanned, which will cause differences between the constructed map and the actual environment.

To quantitatively analyze the map's location accuracy, we compare and analyze the ground truth (GPS measurement values), the estimated value of the proposed algorithm, and the dead reckoning value.

Since the GPS measured value is the latitude and longitude information, we used the geodesy package provided by ROS in the experiment and completed the conversion of latitude and longitude coordinates to two-dimensional coordinates. In this way, we unified the above three coordinate values under the same reference system to evaluate the SLAM algorithm's positioning accuracy.

**Figure 14:**
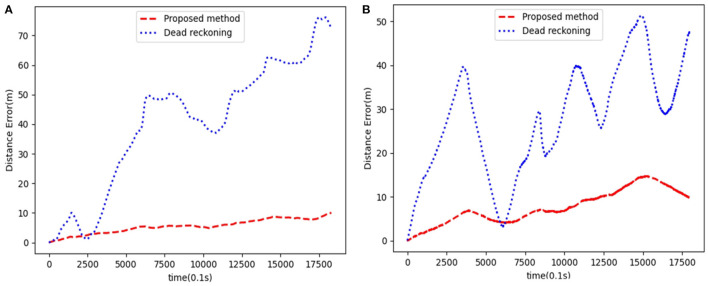
Panel **(A)** is the relative localization errors among the dead-reckoning trajectories, the proposed SLAM algorithm and the ground truth using data from scene1. Panel **(B)** is the scene2.

The trajectory result graph is shown in Figures 12B, 13B, 14 shows the relative localization errors. From Figure 14 we can find that with the accumulation of time, the deviation of the odometer will gradually increase. Using SLAM for simultaneous positioning and mapping will correct the odometer's deviation, correct the pose, and build a better map.

The maps based on the multi-beam sonar SLAM algorithm proposed in this paper can better represent the environment's characteristics. And positioning accuracy is also better than pure dead reckoning.

## 5. Conclusions

This paper proposed a SLAM algorithm using MFLS. Two problems are solved: Aiming at the slow processing speed caused by a large amount of MFLS image data, and a method is proposed to convert the collected sonar image into sparse point cloud format data through threshold segmentation and distance-constrained filtering; Based on the proposed method, the DVL, IMU, and MFLS data are fused, and then the RBPF-based SLAM method is used to suppress the accumulation of errors of the inertial unit and generate an accurate occupancy grid map. Finally, we used BlueROV2 as the experimental carrier, conducted field tests in the experimental pool and Liquan Lake, Xi'an, Shaanxi, and achieved good positioning and mapping results.

## 6. Future work

This article mainly uses MFLS image data to be converted into sparse point cloud data format for SLAM experiments, which involves the problem of sonar filtering and data matching after filtering. There are still some problems currently, such as the inability to fully extract environmental features due to clutter interference and the map drifting due to data matching failure. For these problems, future work will continue to study better filtering methods and data matching methods to improve the accuracy of positioning and mapping.

## Data Availability Statement

The original contributions presented in the study are included in the article/supplementary material, further inquiries can be directed to the corresponding author/s.

## Author Contributions

CC and FZ presented the idea in this article and wrote the paper together. FZ and WL provided technical support and experimental equipment. DY and CW helped complete the experimental verification. All authors contributed to the article and approved the submitted version.

## Funding

This study was supported by the National Natural Science Foundation of China (52171322), the National Key Research and Development Program (2020YFB1313200), and the Fundamental Research Funds for the Central Universities (D5000210944).

## Conflict of Interest

The authors declare that the research was conducted in the absence of any commercial or financial relationships that could be construed as a potential conflict of interest.

## Publisher's Note

All claims expressed in this article are solely those of the authors and do not necessarily represent those of their affiliated organizations, or those of the publisher, the editors and the reviewers. Any product that may be evaluated in this article, or claim that may be made by its manufacturer, is not guaranteed or endorsed by the publisher.
